# The Influence of Lifestyle and Treatment on Oxidative Stress and Inflammation in Diabetes

**DOI:** 10.3390/ijms232415743

**Published:** 2022-12-12

**Authors:** Magdalena Wronka, Julia Krzemińska, Ewelina Młynarska, Jacek Rysz, Beata Franczyk

**Affiliations:** 1 Department of Nephrocardiology, Medical University of Lodz, ul. Zeromskiego 113, 90-549 Lodz, Poland; 2 Department of Nephrology, Hypertension and Family Medicine, Medical University of Lodz, ul. Zeromskiego 113, 90-549 Lodz, Poland

**Keywords:** oxidative stress, inflammation, diabetes, physical activity, supplementation

## Abstract

Diabetes is considered a new pandemic of the modern world, and the number of sufferers is steadily increasing. Sustained hyperglycemia promotes the production of free radicals and leads to persistent, low-grade inflammation. Oxidative stress causes mitochondrial destruction, which along with activation of the hexosamine pathway, nuclear factor-κB (Nf-κb), p38 mitogen-activated protein kinase (p38 MAPK), c-jun NH2 terminal kinase/stress-activated protein kinase (JNK/SAPK) or toll-like receptors (TLRs), leads to pancreatic β-cell dysfunction. However, there is also the protective mechanism that counteracts oxidative stress and inflammation in diabetes, mitophagy, which is a mitochondrial autophagy. An important part of the strategy to control diabetes is to lead a healthy lifestyle based on, among other things, regular physical activity, giving up smoking, eating a balanced diet containing ingredients with antioxidant potential, including vegetables and fruits, and using hypoglycemic pharmacotherapy. Tobacco smoke is a recognized modifiable risk factor for many diseases including diabetes, and it has been shown that the risk of the disease increases in proportion to the intensity of smoking. Physical activity as another component of therapy can effectively reduce glucose fluctuations, and high intensity interval exercise appears to have the most beneficial effect. A proper diet not only increases cellular sensitivity to insulin, but is also able to reduce inflammation and oxidative stress. Pharmacotherapy for diabetes can also affect oxidative stress and inflammation. Some oral drugs, such as metformin, pioglitazone, vildagliptin, liraglutide, and exenatide, cause a reduction in markers of oxidative stress and/or inflammation, while the new drug Imeglimin reverses pancreatic β-cell dysfunction. In studies of sitagliptin, vildagliptin and exenatide, beneficial effects on oxidative stress and inflammation were achieved by, among other things, reducing glycemic excursions. For insulin therapy, no corresponding correlation was observed. Insulin did not reduce oxidative stress parameters. There was no correlation between glucose variability and oxidative stress in patients on insulin therapy. The data used in this study were obtained by searching PubMed online databases, taking into account recent studies.

## 1. Introduction

Diabetes is a metabolic disease characterized by inadequate glycemic control [1]. It can be diagnosed when one of the following criteria is met: fasting plasma glucose ≥126 mg/dL (7.0 mmol/L); plasma glucose ≥200 mg/dL (11.1 mmol/L), along with symptoms of hyperglycemia; plasma glucose ≥200 mg/dL (11.1 mmol/L) 2 h after glucose administration in the course of an oral glucose tolerance test (OGTT); glycated haemoglobin A1c (HbA1c) ≥6.5% [2]. The cause, clinical manifestation and treatment of diabetes depends on its type [1]. The following types of diabetes are distinguished: type 1 diabetes (T1DM); type 2 diabetes (T2DM); as well as other specific types of diabetes (e.g., monogenic diabetic syndromes); gestational diabetes [2]; steroid-induced diabetes; and others [1].

The treatment of patients with T1DM is based on insulin therapy, while the treatment of T2DM includes both oral drug therapy (e.g., biguanides, sulfonylureas, α-glucosidase inhibitors) and insulin administration [1,3]. In patients with T2DM, especially in the early stages, lifestyle changes, primarily the treatment of overweight/obesity, play a major role in controlling the disease [1,4]. To that end, physical activity, diet, behavioral therapy and, if needed, pharmacotherapy and even surgical treatment of obesity are recommended [4].

The goal of diabetes management is to maintain HbA1c at <7.0% for adults [5] and children [6], <7.0–7.5% (exceptionally <8.0%) for older adults [7], and <6.0% (exceptionally <7.0%) for pregnant women [8]. This is important due to the significance of preventing or delaying the development of diabetes complications [9].

Oxidative stress and inflammation play a major role in the pathogenesis of diabetes. The low antioxidant capacity of pancreatic β-cells results in pancreatic cell failure in both T1DM and T2DM [10,11]. T1DM has an autoimmune basis, resulting in a number of processes that lead to inflammation [11,12]. In T1DM, activated macrophages produce reactive oxygen species (ROS) and pro-inflammatory cytokines that inhibit mitochondrial function and damage pancreatic β-cells [11]. Oxidative stress contributes to insulin resistance and, ultimately, T2DM through multiple mechanisms [10] including, but not limited to, the dysfunction [10,12,13] and apoptosis of pancreatic β-cells [10,13]. Sustained hyperglycemia further predisposes to the production of reactive oxygen and nitrogen species (RONS), resulting in the accumulation of free radicals [12,14]. To prevent the accumulation of ROS, antioxidant intervention is required to control oxidative stress [12].

In the case of T2DM, prevention based primarily on lifestyle changes is essential [15]. A key element in the treatment and prevention of diabetes is regular physical activity, which can contribute to a reduction in oxidative stress and the improvement of insulin sensitivity, both of which are processes that play a key role in the pathogenesis of diabetes [16,17]. It has been shown that 150 min of physical activity per week, with the addition of losing 7% of the original body weight and maintaining that weight, reduces the risk of the disease by 58% over three years. Physical activity alone improves insulin sensitivity and reduces the risk of developing T2DM by 44% [15]. A single moderate-intensity workout is able to significantly increase glucose uptake by cells through an increase in glucose transporter type 4 (GLUT-4). Unfortunately, the inconvenience associated with physical activity in patients with T2DM is the increased incidence of hypoglycemia and significant cardiovascular burden. However, moderate aerobic or stretching exercises have been shown to benefit the cardiovascular system and glycemic control in diabetes. The fact is that the selection of appropriate exercise for obese patients with T2DM is problematic, and its intensity and frequency should be chosen carefully and on an individual basis [18,19].

Nutrition can also be highly effective in terms of metabolic control among patients with diabetes; thus, many researchers have attempted to determine which dietary components have the most beneficial effects [20]. Clinical improvement in patients with T2DM can be achieved by increasing antioxidant defenses through consuming supplements with antioxidant potential or by including more fruits and vegetables in the diet [21]. Vitamin C and polyphenols are substances of natural origin, of which vegetables and fruits are a particularly rich source; a diet supplemented with these substances can effectively protect against the consequences of oxidative stress and thus reduce the risk of developing diabetes and other cardiovascular complications [22,23]. A high-protein diet also has a number of beneficial properties, including effectively improving the lipid profile, glycemic control, and lowering the blood pressure, and it therefore appears that it may be effective in both the prevention and treatment of diabetes [20]. The combination of physical activity and an appropriate diet is able to delay, or even inhibit, the progression of the pre-diabetic state to overt T2DM [16].

## 2. Oxidative Stress—Basic Information

**Figure 1:**
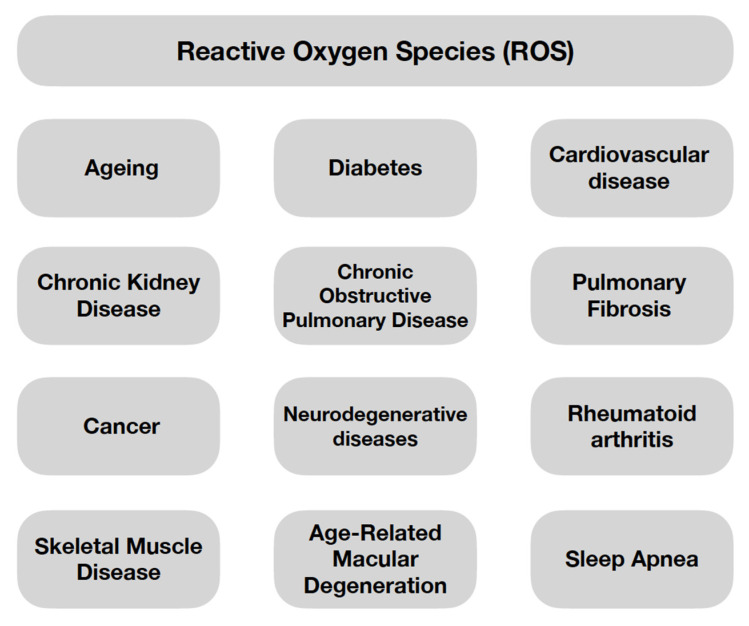
The Examples of Processes/Diseases Caused by Oxidative Stress [24,25].

The disproportion involving the predominance of prooxidant ROS over antioxidant substances (both enzymatic and non-enzymatic) is called oxidative stress [24,25,26,27]. This phenomenon underlies the ageing process, as well as the development of many diseases, including diabetes, cardiovascular disease (CVD), cancer and others, shown in Figure 1 [24,25]. It is caused by excessive concentrations of ROS, which have damaging effects on proteins, lipids and nucleic acids through oxidation, thereby leading to cell destruction and subsequent disorders, which in turn lead to disease [24,25]. There is a strong link between the ageing process and oxidative stress and inflammation, and these phenomena intensify with age [14,28,29,30,31]. Moreover, excess ROS accelerates the rate of ageing [32]. As we age, our antioxidant mechanisms weaken, rendering the body more susceptible to oxidative stress. In addition, the repair and adaptive mechanisms of the ageing body deteriorate [29].

**Figure 2:**
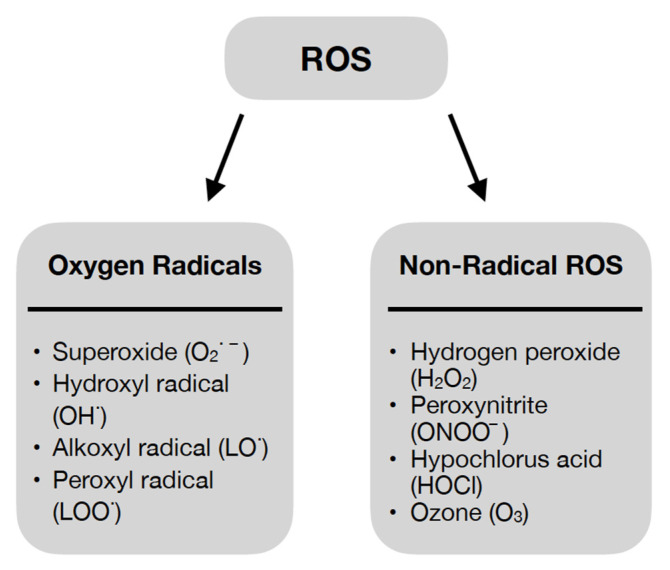
Oxygen Radicals and Non-Radical Reactive Oxygen Species [27].

ROS are highly reactive derivatives of molecular oxygen. They owe their properties to an unpaired electron on the valence shell [24]; however, the role of ROS is not limited to destructive action against cellular components [24,25]. They have been shown to be involved in physiological processes of the body in small amounts [24,27]. ROS also have functions in regulating the signaling pathways responsible for many cellular processes such as proliferation and survival—involving mitogen-activated protein kinase (MAPK) or apoptosis and ageing—involving p66 Src homology/collagen (Shc) [25]. Thus, the profile of the effect of ROS on the organism—negative or positive—is dependent on its concentration [24]. ROS can be divided into oxygen radicals and non-radical ROS, as shown in Figure 2 [27].

Approximately 90% of endogenous ROS are produced in the mitochondria through electron leakage from the respiratory chain, ultimately leading to the formation of O_2_^·−^, which is a precursor to other ROS. The second location where ROS are produced in the body are in the phagocytic cells responsible for the respiratory burst. Infection by bacteria/fungi/parasites results in the activation of phagocytic cells. With the involvement of nicotinamide adenine dinucleotide phosphate (NADPH) oxidase, a respiratory burst occurs. As a result, O_2_^·−^ is produced, followed by other ROS. Ultimately, bacteria/fungi/parasites are eradicated. To a lesser extent, peroxisomes and the endoplasmic reticulum are also responsible for endogenous ROS production [24].

A thorough understanding of the mechanisms of ROS action is crucial for the comprehension of the pathogenesis of diseases underlying oxidative stress. In addition, this understanding allows for the precise identification of therapeutic targets [27].

### Oxidative Stress and Inflammation—The Role in the Development of Diseases

The basis of the pathogenesis of diabetes, as well as the formation of its complications, is oxidative stress [26] and chronic, low-grade inflammation [10,33,34]. Persistent hyperglycemia promotes the production of free radicals [33] (mainly O_2_^·−^) in β-cells through the mitochondrial respiratory chain, as well as NADPH oxidase [10]. Hyperglycemia also leads to persistent inflammation, expressed by elevated levels of pro-inflammatory cytokines and inflammatory markers, e.g., tumor necrosis factor α (TNF-α) and C-reactive protein (CRP) [33]. Chronic, low-grade inflammation leads to insulin resistance and increased glycemia, which further leads to the development of T2DM [34]. Using 8-epi-prostaglandin F2α (8-epi-PGF2α), it has been shown that there is a correlation between the occurrence of insulin resistance and oxidative stress [33]. The pathomechanism of insulin resistance, related to oxidative stress, is based on several molecular mechanisms [10], which will be described below. Oxidative stress is predisposed to decreased insulin production caused by the inhibition of the nuclear transcription factors Pdx-1 (insulin promoter factor 1) and MafA (a transcription factor) [10,12], which impairs insulin secretion [10,12,13] by opening the K_ATP_ channels [10,12] and inhibiting calcium flow [12], and also has a proapoptotic effect on β-cells [10,13]. Pancreatic β-cell dysfunction is caused by, among other things, the destruction of mitochondria by oxidative stress [10,12,13], as well as the activation of the: hexosamine pathway, nuclear factor-κB (Nf-κb), p38 mitogen-activated protein kinase (p38 MAPK), c-jun NH2 terminal kinase/stress-activated protein kinase (JNK/SAPK) or toll-like receptors (TLRs) [10]. Another cause of insulin resistance and the development of diabetes related to oxidative stress is the reduced expression of the GLUT-4 receptor, on which glucose entry and insulin sensitivity of the adipose tissue cells, cardiomyocytes and myocytes, depend. Excess free radicals cause fewer GLUT-4 receptors to localize to the cell membrane [10,35]. In addition, mitochondrial damage caused by oxidative stress results in a lack of the energy required for glucose uptake, which further exacerbates insulin resistance [10]. Free radicals also cause the impairment of the insulin signal transduction pathway [10,12]. This occurs at multiple levels, among which can be found the activation of the p38 MAPK [10,12], c-jun NH2 terminal kinase (JNK) and IκB kinase β (IKKβ)/NF-κb [12] pathways, which causes serine phosphorylation of (insulin receptor substrate-1) IRS-1 and (insulin receptor substrate-2) IRS-2 [10,12]. In T1DM, immune-activated macrophages, among others, are involved in the destruction of pancreatic beta cells. They produce ROS (e.g., O_2_^·−^, H_2_O_2_, NO) and pro-inflammatory cytokines, including interleukin 1 (IL-1), which is a potent activator of the JNK pathway [11]. Derivatives of reactive oxygen metabolites (d-ROMs)-markers of oxidative stress are considered to be reliable indicators of oxidative stress. Its levels were measured in a study by Morandi et al. [36]. This showed that oxidative stress is significantly higher in children with T1DM, compared to healthy children [36].

An excess of RONS with associated reduced cellular antioxidant capacity also lies on the basis of diabetic complications, both micro- and macro-vascular. The mitochondrial overproduction of ROS leads to the activation of the advanced glycation end products (AGEs) molecular pathway, among others, resulting in the increased production of pro-inflammatory cytokines and growth factors [14,37]. Further molecular pathways activated in hyperglycemia include polyol pathway flux, hexosamine pathway flux and protein kinase C activation [14,37,38]. This results in vasoconstriction, tissue ischemia [38] and, consequently, abnormal angiogenesis [37]. Moreover, oxidative stress has negative effects on Schwann cells, myelinated axons and sensory neurons. The disruption of the axon transport function leads to diabetic neuropathy. Hence, it can be concluded that oxidative stress also plays an essential role in impaired wound healing in diabetic patients [38]. The complications of diabetes lead to disability and even death in patients. Therefore, it is important to have a thorough understanding of the pathomechanisms of the effects of ROS on the development of diabetes and targeted antioxidant treatment [12].

## 3. Molecular Mechanisms to Counteract Oxidative Stress and Inflammation in Diabetes

Focusing on the mechanisms to counteract oxidative stress and inflammation in diabetes, it is important to mention mitophagy, which is mitochondrial autophagy [13,39]. Mitophagy is a process activated in response to free radicals and pro-inflammatory cytokines [13]. Oxidative stress damages mitochondrial DNA (mtDNA), which leads to the activation of proapoptotic pathways, resulting in β-cell failure and hyperglycemia [13,40]. Mitophagy removes dysfunctional mitochondria, preventing an increase in ROS levels [13,39]. Thus, it counteracts the damage caused by oxidative stress and inflammation, thereby protecting β-cells from apoptosis. The gene CLEC16A, known as the T1DM susceptibility gene, is associated with the process of mitophagy. CLEC16A, through ubiquitin E3 ligase, controls mitophagy. The overexpression of the T1DM susceptibility gene is thought to be an essential factor responsible for reducing inflammation-induced β-cell apoptosis [13]. Shu et al. [40] also highlight the role of the mitophagy receptor ATAD3B. Increasing ATAD3B activity results in the removal of mtDNA damaged by oxidative stress, so it may be a therapeutic target. Mitophagy also plays an important role in maintaining redox homeostasis in the course of many diseases [39,41,42]. In addition to diabetes, we can include CVD [41] and neurodegenerative diseases [42], among others. As mitochondria play a key role in the cardiovascular system, the development of CVD e.g., atherosclerosis, myocardial infarction or various types of cardiomyopathy, may depend on the mitophagy process [41]. Moreover, the dysregulation of mitophagy has been shown to be a key factor in the development of neurodegenerative diseases e.g., Alzheimer’s, Parkinson’s or Huntington’s disease [39,42].

## 4. Effects of Physical Activity on Glycemic Control, Oxidative Stress Biomarkers and Inflammation

Regular physical activity is an important part of the diabetes management strategy, providing many benefits, such as weight loss and improvement in Body Mass Index (BMI), a reduction in inflammatory parameters and oxidative stress biomarkers, increased tissue sensitivity to insulin, as well as the improved quality of life and well-being of patients. Regular exercises have been shown to increase the release of anti-inflammatory cytokines and they lead to a reduction in pro-inflammatory cytokines, e.g., TNF-α and interleukin 1β (IL-1β), interleukin 6 (IL-6), interleukin 18 (IL-18), CRP, which play a role in the pathogenesis of diabetes [16].

Chen et al. [18] conducted a randomized study evaluating the effects of simplified t’ai chi exercises (TCE) on inflammatory markers, oxidative stress parameters and glycemic control in obese patients with T2DM, among others, who were assigned to either the TCE or conventional exercise (CE) group. After 12 weeks, no improvement in HbA1c was seen in the TCE group; however, there were a marked improvement in BMI, high-sensitivity C-reactive protein (hsCRP) and malondialdehyde (MDA), compared to baseline values. In contrast, the CE group showed no improvement in any of the aforementioned parameters. Although their metabolic status and physical condition improved with the use of TCE, no increase in insulin sensitivity was observed [18]. In contrast, Gouveia et al. [19] studied how another type of exercise, pilates, affects oxidative stress and metabolic control in patients with T2DM. After eight weeks of exercise, a decrease in HbA1c and MDA was observed in the intervention group, finding a positive correlation between HbA1c and oxidative stress [19]. Farabi et al. [17] focused on another aspect and conducted a study to evaluate the effect of a single round of moderate-intensity aerobic exercise on daily glycemic variability and oxidative stress in obese patients with impaired glucose tolerance or diabetes. Physical activity was shown to have a significant effect on the correlation between nocturnal glycemic variability and diurnal oxidative stress. It was also shown that even a single workout performed in the morning is able to reduce glycemic fluctuations and reduce urinary isoprostane-15 excretion in this group of patients. An interesting association is the observed correlation of reduced levels of isoprostane-15 in the daytime urine collection after moderate exercise with a subsequent reduction in day and night glycemic variability [17].

It is known that chronic physical activity reduces ROS production, increases antioxidant potential, and enhances cellular sensitivity to insulin; however, it has not been determined which level of exercise intensity has the best antioxidant and anti-inflammatory potential. Mallard et al. [43] focused on this problem by conducting a study involving 36 patients with T2DM, who were assigned to two groups: one group performing high-intensity interval exercise and one group performing moderate sustained exercise. Despite the use of physical activity of different intensities, no significant difference was observed in the levels of markers of oxidative stress and inflammation after 12 weeks of intervention and one year of follow-up between the groups, however, there was also no increase in these markers. In contrast, the maintenance of total antioxidant capacity was observed in a group of individuals performing high-intensity interval training for a year. Given the differences in protein carbonyls between men and women, it has been suggested that interventions involving physical activity may have different effects depending on gender [43]. A similar study was conducted by Karstoft et al. [44], who also evaluated the relationship between interval training or continuous gait training and oxidative stress, glycemic control and variability in patients with T2DM. During the study, a significant reduction in fasting glucose was noted in the group with interval training compared to the placebo group, and a downward trend in mean glucose concentration or time in hyperglycemia was also noted. In addition, a reduction in mean and maximum glucose concentrations in continuous glucose monitoring was observed in the same group, regardless of the level of weight reduction. However, none of the training interventions resulted in changes in oxidative stress markers. Surprisingly, an exercise consisting of continuous gait training did not improve glycemic control and, thus, the metabolic profile. The study did not observe a correlation between glycemic levels such as mean or maximum glucose levels and oxidative stress [44]. On the other hand, Farinha et al. [45] made a comparison between intensive interval training and strength training on glycemic control, oxidative stress markers and inflammation, this time in patients with T1DM. Intensive training improved glycemic control (fasting glucose and HbA1c) and antioxidant potential (total antioxidant capacity (TAC), catalase (CAT), Manganese-dependent SOD), while it did not reduce inflammatory biomarkers. Despite the fact that HbA1c levels decreased slightly (−0.26%), they have a significant impact on patient mortality (10% decrease). Regardless of the type of intervention, high-intensity physical activity has been shown to positively affect glycemic control and antioxidant potential, while it does not improve inflammatory biomarkers. It has also been shown that intensified activity-interval + strength training is required to reduce insulin intake in patients with T1DM [45]. A study by Hoseini et al. [46] examined the effects of aerobic training on markers of oxidative stress and inflammation in patients with t2 diabetes. They found that an eight week intervention in the form of AT activity positively influenced glycemic control, most likely through a mechanism of increased antioxidant defense and decreased inflammatory markers (IL-1β, hs-CRP, TNF-α, interferon-γ (IFN-γ)), and increased the production of anti-inflammatory cytokines (increase in IL-4). Due to the small number of participants, conclusions should be made cautiously; however, it seems that physical activity, particularly when combined with vitamin D supplementation, is able to reduce biomarkers of oxidative stress and inflammation in patients with T2DM [46].

The effects of some of the listed types of physical activity on oxidative stress and inflammation are shown in Table 1 [17,18,19,43,44].

## 5. Effect of Cigarette Smoking on the Development of Type 2 Diabetes

Tobacco smoke contains a number of harmful compounds that can have deleterious effects on almost all organs and contribute to the development of many diseases, including T2DM [47,48,49]. Cigarette smoking can cause disease through a mechanism of increased systemic inflammation and free radical production [48]. Smokers have been shown to have a higher risk of developing T2DM than the nonsmoking population by up to 30–50%, and this risk is even higher in heavy smokers [49].

In a meta-analysis conducted by Akter et al. [47], the aim was to determine the relationship between smoking status and the risk of developing T2DM in the Japanese population. They found an increased risk of developing T2DM in active smokers (RR = 1.38) and former smokers (RR = 1.19) compared to non-smokers, with a similar distribution in both sexes. Moreover, they discovered a linear increase in the risk of developing T2DM correlating with the increasing number of cigarettes smoked. Interestingly, every additional ten cigarettes smoked per day was shown to increase the risk of the disease by 16%. It is noteworthy that this risk decreases with smoking-free time, reaching similar values to never-smokers after ten years of abstinence. However, given the tendency for weight gain after smoking cessation to predispose to the development of T2DM, further studies should be conducted on whether short-term non-smoking can increase the risk of developing the disease [47]. Grøndahl et al. [49] investigated how heavy smoking would affect postprandial glucose metabolism and noted that fasting glucose levels reached slightly higher values in smokers than in non-smokers; in addition, smokers had high fasting glucagon levels, which may be a risk factor for developing T2DM. On the other hand, during the 2 h after meal intake, smokers who did not smoke during the per-meal period showed higher glycemic values than non-smokers. Interestingly, glycemic fluctuations and peak glycemic values after the meal were lower when smokers smoked, but this was likely due to the delay in gastric emptying caused by smoking [49]. A meta-analysis by Yuan et al. [50] evaluated the relationship between gender and the risk of T2DM in smokers. They observed that actively smoking women had a 27% and smoking men a 35% risk of developing the disease compared to non-smokers, while the risk of developing T2DM in those who quit smoking compared to never smokers was 11% higher equally in both sexes. This habit was shown to induce a similar risk of disease in both men and women and confirmed the relationship between smoking and the development of T2DM. Importantly, it was also observed that smoking cessation positively reduced the risk of diabetes to the same extent in both groups. A limitation of the meta-analysis was the inclusion of past smokers in some studies in the non-smoking group; however, this did not significantly affect the results [50]. Another topic was addressed by Wang et al. [51], who evaluated the association between smoking during pregnancy and the development of gestational diabetes. It has been shown that cigarette smoking during pregnancy, regardless of its intensity, does not correlate with a higher risk of developing gestational diabetes. However, this meta-analysis has several weaknesses, including that the selected studies were self-reported by women and there are few additional studies on the topic [51].

## 6. Effects of Diet and Supplementation on Oxidative Stress and Inflammation and Their Correlation with Glycemic Control

### 6.1. Vitamin C

Impaired glucose metabolism, insulin resistance and vascular complications have been shown to result from increased oxidative stress and excess ROS in patients with T2DM, and it has been speculated that antioxidant therapy may be beneficial in these patients. One antioxidant that could have applications in improving glycemic control and reducing cardiometabolic risk is water-soluble vitamin C [52]. It has been suggested that the chronic intake of vitamin C in high doses (≥1 g/day) may improve glucose metabolism and increase insulin sensitivity in people with T2DM [53].

Mason et al. [52] conducted a meta-analysis to evaluate the effectiveness of vitamin C supplementation in reducing oxidative stress and controlling glycemic variability in patients with T2DM. The study found that vitamin C could have a beneficial effect on the course of the disease by lowering HbA1c, fasting blood glucose, postprandial blood glucose and oxidative stress markers such as MDA, among others. However, due to the heterogeneity between studies and the imprecision of the results, this conclusion can be considered hesitant. Moreover, a factor limiting the reliability of the results is that the studies tended to be short (<6 months) and were conducted on too few participants. It appears that vitamin C may be an inexpensive and readily available adjunct to the treatment of T2DM; however, further studies conducted for a longer duration with larger numbers of patients are needed. Mason et al. [52] suggest that those taking vitamin C regularly and those with higher HbA1C in the study may benefit most from supplementation [52]. Another study, also conducted by Mason et al. [53], examined the effects of high-dose ascorbic acid (AA) supplementation on skeletal muscle insulin sensitivity and markers of oxidative stress in the same group of patients. This study showed that the use of high doses of AA led to a significant improvement, up to 60%, in insulin-stimulated glucose disposal delta rate of glucose disappearance (ΔRd) during the four month supplementation. An increase in insulin sensitivity in the peripheral tissues was also observed, likely due to increased glucose utilization in the skeletal muscle. In addition, increased AA concentrations were noted in skeletal muscle, which in effect increases antioxidant potential during hyperinsulinemia and improves insulin sensitivity in these tissues. It has been suggested that the increase in AA concentration in muscle may be due to an increase in the expression of sodium-ascorbate co-transporter 2 (SVCT2) protein, which facilitates AA accumulation in skeletal muscle, as observed by Mason et al. [53]. The results of this study suggest that high-dose, chronic AA supplementation may be an important component and adjunct of T2DM therapy [53]. In contrast, a study by Ragheb et al. [22], which aimed to evaluate the effect of supplementation with vitamin C or vitamin C in combination with rutin in the T2DM, showed no significant changes in the levels of HbA1c, fasting insulin, Homeostatic Model Assessment—Insulin Resistance (HOMA-IR) and oxidative stress markers—MDA or SOD between the two groups at the end of the study. However, a decreasing trend was noted in the HbA1c and fasting insulin levels [22].

### 6.2. Vitamin E

Patients with T2DM are at risk of accelerated atherosclerosis due to elevated rates of inflammation and increased oxidative stress. It appears that vitamin E, particularly its most bioavailable form, tocopherol, may provide a dietary component or supplement with antioxidant and inflammation-reducing properties [54]. Vitamin E is a lipophilic compound that reduces oxidative stress by inhibiting lipid peroxidation. Its action can be supported by other vitamins, such as, vitamin C regenerates vitamin E, while B-carotene assists alpha-tocopherol in reducing lipid peroxidation to a greater extent than the action of each vitamin individually. Thus, it is noteworthy that the supplementation of several antioxidant vitamins exerts a greater synergistic protective effect than the use of each vitamin alone [55].

Wu et al. [54] conducted a randomized six week study evaluating supplementation with alpha tocopherol or mixed tocopherols on the incidence of inflammation and oxidative stress in patients with T2DM. A marked decrease in plasma F2-IsoPs levels was observed in patients supplementing mixed tocopherols or alphaT, in contrast to those in the placebo groups. However, no significant effect was noted on urinary F2-IsoPs, glutathione peroxidase (GSH-Px) and SOD activities in erythrocytes, as well as blood MPO activity, blood IL-6, hsCRP or TNF-α levels. An interesting finding is that both Talfa and mixed tocopherols show similar antioxidant effects. However, the researchers suggest that while patients with uncontrolled T2DM may benefit from supplementation, those with a well-controlled disease may not benefit significantly from further reductions in oxidative stress through supplementation with Talfa or a tocopherol blend [54]. An interesting study was conducted by de Oliveira et al. [55], which showed that the vitamin complex (β-carotene, ascorbic acid and α-tocopherol) showed distinct effects depending on the dose consumed and on the group of patients, taking into account patients without diabetes and those with type 1 diabetes. It turned out that with lower doses it showed antioxidant properties, reducing ROS production in both groups of patients, while higher doses showed pro-oxidant properties in patients without diabetes. The use of the vitamin complex at higher doses decreased the production of interleukin 4 (IL-4) and TNF-α and increased the production of IL-6 by mononuclear cells in patients without diabetes. Importantly, no changes in interleukin 8 (IL-8) production were observed. These results show that, depending on the dose used, vitamins can inhibit or activate oxidative stress and pro-inflammatory cytokines via signalling pathways [55]. Another topic was addressed by Jamilian et al. [56], who studied the effects of supplementation with omega 3 fatty acids and vitamin E on inflammation and oxidative stress markers in women with gestational diabetes mellitus (GDM). There was a significant increase in NO and TAC levels and a significant decrease in plasma MDA levels in the intervention group compared to the placebo group. Supplementation did not cause significant changes in serum hs-CRP levels. The results suggest that the described supplementation has beneficial effects on reducing oxidative stress, pro-inflammatory cytokines, protection against oxidation and a reduction in lipid peroxide production in GDM patients [56].

All markers of oxidative stress and inflammation that were used in the vitamin C and E studies in this article are listed in Table 2 [22,52,53,54,55,56].

### 6.3. Fruits

The muscat grape is a rich source of polyphenols that possess a number of properties, including antioxidant potential. Banini et al. [57] set out to test whether products made from muscat grapes, such as wine or grape juice, have a cardioprotective effect in patients with T2DM. Patients were assigned to one of three groups: muscat grape (MJ), muscatel grape wine (MW) and non-alcoholic muscat grape wine (Dz-W), while patients without diabetes were assigned to the group consuming MJ or to the control group. It was observed that, in contrast to the other groups, patients with T2DM who were given MJ or MW had a higher fasting insulin/glucose ratio, which is a risk factor for the development of insulin resistance. Elevated levels of vitamin C and E were observed in the diabetic patients supplementing with DZ-W, which may suggest better antioxidant activity. The findings suggest that red grapes have a protective effect on the development of degenerative diseases, including diabetes [57].

Hokayem et al. [58] also conducted a study to evaluate the benefits of grape polyphenols in preventing oxidative stress and insulin resistance in healthy but history-burdened type 2 diabetic patients on a high-fructose diet. After an eight week supplementation with grape polyphenols, a reduction in muscle thiobarbituric acid-reactive substances and F2-IsoPs in urine was observed in the placebo group. No significant changes were seen in oxidative stress indicators such as erythrocyte and muscle SOD, GSH-Px or enzymatic activity of catalase. There were also no significant changes in cytokines such as IL-1α, IL-6, TNF-α, among others. After six days on the high-fructose diet, patients in the placebo group showed a significant increase in urinary F2-IsoPs and an increase in protein carbonylation in muscle, in contrast to the group receiving polyphenols. Another important observation after consuming a high-fructose diet was a decrease in the fasting hepatic insulin sensitivity index tested in the placebo group, compared to the intervention group, in which polyphenols prevented insulin resistance, thus ensuring a stable metabolic state [58]. Hedge et al. [21], on the other hand, evaluated the effect of a three month diet containing two low-calorie fruits per day on glycemic control and of the reduction in oxidative stress in patients with T2DM. During the study, 123 patients were assigned to the expanded diet or placebo group. In the group consuming low-calorie fruit, a significant increase in vitamin C and a decrease in glutathione were observed compared to the baseline values; in addition, HbA1c and mean plasma glucose levels decreased significantly, in contrast to the placebo group. No changes were observed in vitamin E and SOD. The results of the study not only provided important information, but disproved the notion that patients with diabetes should not consume fruit [21]. A randomized study by Hsia et al. [23] focused on evaluating whether daily supplementation with a low-calorie cranberry drink would affect insulin sensitivity, oxidative stress and some cardiovascular risk factors in obese patients with high glycemia or abnormal glucose tolerance. Patients were divided into two groups: a cranberry drink supplement group and a placebo group. During the study, there were no statistically significant differences in the glucose utilization rate and fasting glucose and 2 h glucose concentration from baseline in the OGTT test between the two groups. The components of the lipidogram included also showed no differences during the study; only the percentage concentration of triglycerides (TG) decreased from baseline in the supplement group, while it increased in the placebo group. A significant difference was also noted in the urinary concentration of 8-isoprostanes. It is noteworthy that there were no differences in inflammatory marker CRP concentrations from the baseline after the cranberry drink, but considering baseline CRP concentrations, as the subgroup of patients with high CRP (>4 mg/L) showed a significant decrease in percentage TG concentrations from the baseline, in contrast to the placebo group. Based on these results, it appears that patients with elevated inflammation may benefit more from cranberry beverage supplementation [23]. Sohrab et al. [59], in turn, evaluated the effect of pomegranate juice (PJ) intake on markers of oxidative stress in patients with T2DM. The study included 60 patients who were assigned to an intervention group or a control group. The study showed that the six week consumption of 200 mL of PJ by patients with T2DM significantly improved biomarkers of oxidative stress, including an apparent increase in the arylesterase activity of paraoxonase (PON1) and TAC, as well as a marked reduction in oxLDL antibodies and serum oxLDL levels. No negative effect of supplementation on blood glucose levels was noted [59].

It has been suggested that free radicals may play a role in triggering the autoimmune response involved in the destruction of pancreatic islet B cells in T1DM. Nemes-Nagy et al. [60] conducted a study evaluating the effect of blueberry and sea buckthorn concentrate treatment on glycemic control and oxidative stress in children with T1DM. In total, 30 children were included in the study and assigned to an intervention or placebo group. After two months of supplementation, there was a significant increase in erythrocytic Cu/Zn SOD and a reduction in glycemia and HbA1c in the intervention group. However, no favorable correlation was observed between HbA1c levels and SOD and GSH-Px enzyme activity. On the other hand, a side effect of the therapy was hypoglycemia, occurring with greater frequency, which required the optimization of insulin dosage. However, a positive result of this is that the insulin doses had to be reduced in as many as 66.7% of patients due to the reduction in insulin demand that the therapy in question generated. A spectacular effect was also noted with regard to endogenous insulin production, as after two months of supplementation, the value of C-peptide even rose to the age-specific reference level. The results of the study provide us with evidence that blueberry and sea buckthorn concentrate increases antioxidant activity and exerts the regenerative effects on pancreatic B cells in children with T1DM [60].

Table 3 shows the effects of the discussed fruits on oxidative stress and inflammation [21,23,57,58,59,60].

### 6.4. Animal Protein, Vegetable Protein, Eggs and Citrulline

McDonald et al. [61] conducted a study in which they evaluated the effect of replacing glucose in a meal with egg whites or whole eggs on the severity of oxidative stress in men with prediabetes and proved correlations between postprandial glycemia and the severity of oxidative stress. During the study, a test meal enriched with egg whites or whole eggs reduced the duration of elevated MDA, thereby reducing lipid peroxidation, lowering NO levels and showing a vasoprotective effect. It seems that replacing carbohydrate meals with a diet containing egg whites or whole eggs can effectively counteract oxidative stress and reduce CVD risk [61]. Another study, also conducted by McDonald et al. [62], evaluated the timing of the post-meal increase in plasma insulin and showed that a meal containing whole eggs or egg whites was followed by a less intense increase in insulinemia and blood glucose compared to the group consuming a meal with glucose, which translates into a vasoprotective effect, and the whole process is aided by the slowed gastric passage, caused by the increase in cholecystokinin (CCK) activity. No significant benefit was noted with regard to postprandial plasma lipid content [62]. A noteworthy study was conducted by Fuller et al. [63], which aimed to determine how a high egg diet (≥12 eggs/week) affects cardiometabolic risk in patients with prediabetes or T2DM compared to a low egg diet (<2 eggs/week). The study found no important differences in the changes in fasting serum high density lipoprotein cholesterol (HDL-C), LDL cholesterol, TC, TG, apolipoprotein B (apoB), selectin, F2-IsoPs and IL-6 between the two groups. The glycemic control indices were also not significantly different between the two groups. The main finding of this study is that the use of a high-protein diet for 12 months (with a three month weight loss period) in patients with prediabetes or type 2 diabetes did not cause adverse changes in oxidative stress, inflammation, lipidogram or glycemic control, and therefore did not produce deleterious cardiometabolic effects. It is noteworthy that a diet containing a large number of eggs may therefore be as safe in patients with T2DM as in healthy individuals and, importantly, does not increase cardiovascular risk [63]. On the other hand, Pivovarova-Ramich et al. [20] conducted a study to evaluate a diet rich in animal protein (AP) or plant protein (PP) in patients with T2DM. Patients were assigned to a group consuming a diet rich in AP or PP. During the study, it was observed that both diets slightly reduced body fat and BMI; in addition, a significant reduction in fasting glucose, HbA1c levels and increased insulin sensitivity was noted in the AP group. Among inflammatory markers, a reduction in CRP was noted among the AP group and a reduction in TNF-α in the PP group, while in terms of oxidative stress markers, reductions in MDA and protein carbonyls were noted in both groups [20]. Another interesting issue was addressed by Azizi et al. [64], who conducted a study evaluating the effect of alpha-amino acid-citrulline supplementation on oxidative stress and (nitrite/nitrate) NOx in type 2 diabetic patients. At two months, in terms of oxidative stress markers, a marked reduction in serum MDA and fasting blood sugar (FBS) levels and a significant increase in NOx, citrulline and TAC levels were observed compared, to the baseline values, in the citrulline-supplemented group. No significant differences were noted with regard to SOD and GSH-Px. In conclusion, citrulline as a precursor of arginine to increase its bioavailability for NO synthesis seems to be a helpful adjunctive therapy, however, further studies are needed [64].

## 7. Pharmacological Therapy of Diabetes Mellitus and Its Effects on Oxidative Stress and Inflammation

In some cases of T2DM, lifestyle modification may not be sufficient and pharmacological treatment must be implemented. The pharmacological treatment of T2DM is based on both oral medications and insulin therapy, while the treatment of T1DM involves the administration of insulin in the form of multiple daily injections or via an insulin pump [1,3]. The groups of oral drugs used in T2DM include biguanides, sulfonylureas, α-glucosidase inhibitors, thiazolidinediones, dipeptidyl peptidase IV (DPP-4) inhibitors, glucagonlike-peptide-1 (GLP-1) agonist, sodium-glucose transporter-2 (SGLT-2) inhibitors [1]. The choice of therapeutics depends on several factors, including comorbidities or patient-centered factors. According to the American Diabetes Association (ADA)’s recommendations, the first-line treatment for T2DM is lifestyle changes and metformin. Depending on the patient’s individual profile and needs, treatment for T2DM can be based on monotherapy or combination therapy [3,65].

### 7.1. Effects of Insulin Administration on Oxidative Stress and Inflammation in Diabetic Patients

Wang et al. [66] evaluated the relationship between initial insulin dose in 60 newly diagnosed patients with T1DM and blood glucose fluctuations and severity of oxidative stress. Patients were randomly assigned to three groups with different initial doses of continuous subcutaneous insulin infusion. It was observed that high doses of insulin caused the fastest drop in blood glucose levels at the beginning of the study; however, after two and three weeks, this difference blurred as the glucose levels were equally lowered with lower, medium and high doses of insulin. There were no differences in the severity of oxidative stress by insulin dose, as urinary 8-iso-prostaglandin F2α (8-iso-PGF2α)/creatinine concentrations did not differ within the groups [66]. The VARIAFIT study also evaluated the effectiveness of a flexible insulin therapy program on glucose variability, a reduction in inflammation and oxidative stress in patients with T1DM. Blood glucose variability was assessed by parameters such as mean amplitude of glycaemic excursions (MAGE), average daily risk range (ADRR) and low blood glucose index (LBGI). The program in question showed efficacy in stabilizing glycemic variability (decrease in ADRR and LBGI) only among patients with the highest baseline parameters. Despite a significant decrease in 11-dehydro-thromboxane B (TXB2) after six months of intervention, other oxidative stress parameters, such as leukotriene E4 (LTE4) and prostaglandin F2 (PGF2), remained stable. Therefore, there was no correlation between glucose variability and oxidative stress in this group of patients [67].

### 7.2. Effects of Oral Antiglycemic Drugs on Oxidative Stress and Inflammation in Diabetic Patients

Metformin is a reference drug used as the first-line treatment in T2DM because it sensitizes to insulin, improves glycemic control and also reduces postprandial glucose levels, making it widely used [68,69]. A randomized trial conducted by Chakraborty et al. [68] was designed to evaluate the effect of metformin treatment on biomarkers of oxidative stress and inflammation in patients with T2DM. After 24 weeks, the metformin treatment group showed a decrease in HbA1c levels, a greater reduction in advanced oxidation protein products formed as a result of oxidative stress, pentosidine, less generation of oxidative stress within white blood cells and also a reduction in inflammation by lowering the CRP index, compared to the placebo group [68]. On the other hand, a randomized study by Bulatova et al. [69] evaluated the effect of metformin treatment combined with therapeutic lifestyle changes compared to lifestyle changes alone on oxidative stress and TNF-α in patients with pre-diabetes or T2DM. After three months, there was a decrease in HbA1c in both groups of patients, while there was no decrease in FBG, and this trend continued after six months. Importantly, the combination therapy led to a significant reduction in oxidative stress, while lifestyle changes alone proved ineffective. A similar relationship was noted for the inflammatory marker TNF-α, which decreased only in the group using metformin with lifestyle changes. These studies confirmed the effective and safe role of metformin in reducing the oxidative stress of inflammatory markers and glycemic control [69]. Pioglitazone is often added to metformin as another treatment regimen to increase its efficacy on T2DM [70]. Mirmiranpour et al. [71] compared the antioxidant and anti-inflammatory potential of metformin with pioglitazone in patients with recently diagnosed T2DM. Pioglitazone proved effective in reducing the levels of the overall oxidative stress markers advanced glycation end products (AGE) and advanced oxidation protein products (AOPP), and caused an increase in the antioxidant reserve marker ferritin reducing ability of plasma (FRAP). Interestingly, the group taking metformin had a greater increase in FRAP, despite a similar effect in improving glycemic control, suggesting that its antioxidant potential may be independent of glycemic control. Pioglitazone, on the other hand, showed better effects on antioxidant enzymes, such as lecithin-cholesterol asyltransferase, which is involved in lipid metabolism [71]. Singh et al. [70] also examined the effects of metformin and pioglitazone on markers of oxidative stress in patients with T2DM who were assigned to one of three groups: metformin group, pioglitazone group or placebo. During the four week intervention, no differences in the hypoglycemic effects of the two drugs were noted. With regard to oxidative stress markers, both metformin and pioglitazone reduced MDA levels, however, pioglitazone showed a better effect. On the other hand, metformin caused a significant increase in SOD, in contrast to pioglitazone. Although both drugs counteract oxidative stress, only metformin shows antioxidant properties. Singh et al. emphasize that the different mechanism in reducing oxidative stress of the two formulations can be used to better reduce insulin resistance by combining these drugs [70]. Kim et al. [72] investigated the efficacy of vildagliptin or glimepiride attached to metformin therapy in reducing oxidative stress and controlling glycemic variability in T2DM patients. In this randomized trial, metformin-treated patients whose glycemic control was not achieved were assigned to a group additionally receiving vildagliptin 50 mg twice daily or to a group receiving glimepiride 2 mg daily. After 12 weeks of intervention, a decrease in fasting glucose levels and the HOMA-IR index was noted after vildagliptin use, compared to the baseline values. Both drugs significantly lowered HbA1c and glucose levels, which were insufficiently lowered by metformin; however, vildagliptin was better than glimepiride at reducing diurnal glucose fluctuations, and had a lower frequency of hypoglycemic incidents. Importantly, there were no differences in oxidative stress as determined by urinary 8-iso-PGF2α concentrations between the two groups. Despite its many advantages, the study also had its limitations, as it involved a small number of patients, and the study duration was also short [72].

A study by Rizzo et al. [73] showed that treatment with sitagliptin or vildagliptin results in a reduction in oxidative stress and inflammation. Interestingly, patients receiving vildagliptin achieved lower levels of nitrotyrosine, as well as IL-6, IL-18, than those taking sitagliptin. It was observed that this is associated with a decrease in MAGE as a reduction in glycemic fluctuations results in a decrease in oxidative stress and inflammatory markers. Hence, it is recommended to pay more attention to improving MAGE in the treatment of T2DM [73]. Another oral drug in diabetic therapy with proven effects in reducing oxidative stress and lowering inflammatory parameters is exenatide. It is a synthetic incretin peptide with properties that mimic the physiological incretin hormone glucagon-like peptide-1 (GLP-1). The effect of exenatide is also seen in reducing glycemia and HbA1c, limiting glycemic spikes, and has a beneficial effect on body weight. As a result, ROS levels and parameters such as hs-CRP and MCP-1 are not increased. Thus, the effect of exenatide on reducing oxidative stress and inflammation is both direct and indirect [74]. Similar conclusions were reached by studying the effects of liraglutide added to metformin treatment. In addition to reducing oxidative stress (reducing lipid peroxidation), an increase in antioxidant activity by increasing the GSH content was observed. It should be kept in mind that this study lacked a control group to show whether the results obtained were due solely to liraglutide [75]. Important to the impact of oxidative stress on the pathophysiology of T2DM is a new drug, Imeglimin. It is an oral antidiabetic drug that increases insulin action in the liver and skeletal muscle and, importantly, reverses pancreatic β-cell dysfunction. The mechanism of action of Imeglimin is based on restoring the balance in the mitochondrial oxidative-phosphorylation chains, the dysfunction of which causes excessive production of ROS. The result is an increase in mtDNA synthesis and, most importantly, a reduction in oxidative stress by decreasing ROS production [76,77].

The effects of exemplary drugs on markers of oxidative stress and inflammation are shown in Table 4.

## 8. Conclusions

In diabetes, oxidative stress leads to reduced insulin production, impaired insulin secretion, β-cell dysfunction and its apoptosis, reduced expression of the GLUT-4 receptor and impairment of the insulin signal transduction pathway. The above mentioned processes, as well as persistent, low-grade inflammation, lead to insulin resistance and T2DM. Excess ROS influences both the formation of diabetes mellitus and its complications, both micro- and macro-vascular, as well as diabetic neuropathy. However, there is a protective mechanism against oxidative stress and inflammation in diabetes, which is mitophagy. It results in the removal of dysfunctional mitochondria, which prevents the accumulation of ROS. A combination of physical activity and a proper diet is an important part of the management strategy for patients with diabetes, as it contributes to increasing cellular sensitivity to insulin. Among exercises, moderate-intensity training based on aerobic and stretching exercises is recommended, however, high-intensity interval training seems to have the most beneficial effect on average daily glucose levels and time in hyperglycemia. Most papers have shown that physical activity has a positive correlation between glycemic levels and oxidative stress. In terms of diet, increasing attention is being paid to natural sources of antioxidants; hence, a diet supplemented with polyphenols, vitamins C and E and fruits can effectively protect against the consequences of oxidative stress and thus reduce the risk of developing diabetes and other cardiovascular complications. Exploring the mechanisms by which oxidative stress and inflammation affect T2DM will result in better therapeutic targeting and antioxidant treatment, as is the case with imeglimin. Further studies would be useful to determine more precisely the correlation between glucose fluctuations and the severity of oxidative stress during pharmacotherapy. It seems that drugs from different groups of oral antidiabetic drugs can counteract oxidative stress through different mechanisms, so it may be beneficial to use combination therapy, as has been shown for metformin and pioglitazone. Insulin only caused a decrease in TXB2, while no decrease in other parameters, such as LTE4 and PGF2, was observed. Further studies are suggested. Factors limiting the reliability of the results are that the studies were conducted on a small number of participants, the duration of many of the studies was too short, and some of the studies were inaccurate, with no control trials.

## Figures and Tables

**Table 1:** Effects of Specific Activities on Oxidative Stress and Inflammation [17,18,19,43,44].

Authors	Farabi et al. [17]	Chen et al. [18]	Gouveia et al. [19]	Mallard et al. [43]	Karstoft et al. [44]
**Study design**	Randomized controlled trial	Randomized controlled trial	Randomized controlled trial	Randomized controlled trial	Randomized controlled trial
**All patients**	37	104	44	36	14
**Patient category**	Patients aged 18–55 years, with type 2 diabetes with a maximum duration of 7 years, not physically trained	Obese (BMI 30–35) patients aged 40–70 with type 2 diabetes	Patients diagnosed with type 2 diabetes between 44 and 80 years of age	Patients aged between 44 and 65 years, diagnosed with type 2 diabetes in the last 10 years with diastolic dysfunction and exercising less than 210 min/week	Patients with a BMI between 18 and 40 kg/m^2^ with type 2 diabetes mellitus
**Type of activity**	A single set of moderate-intensity exercise lasting 30 min	Simplified t’ai chi exercises	Pilates protocol	High-intensity interval training and moderate-intensity continuous training	Short-term interval walking training and continuous walking training
**Antioxidant/anti-inflammatory effects**	A decrease in the daily concentration of 15-isoprostane F in urine,	Significant decrease in serum hsCRP, decrease in MDA index, no significant changes in PLA2, POX indices	Decrease in CRP levels, decrease in MDA levels	No effect on oxidative stress and inflammation	No effect on oxidative stress, no effect on 8-iso PGF2α levels

BMI, Body Mass Index; hsCRP, high-sensitivity C-reactive protein; MDA, malondialdehyde; PLA2, POX, CRP, C-reactive protein.

**Table 2:** Biomarkers That Have Been Studied to Determine the Antioxidant and/or Anti-inflammatory Properties of a Particular Fruit [22,52,53,54,55,56].

Study Author	Biomarkers of Oxidative Stress or Inflammation
Ragheb et al. [22]	SOD, MDA
Mason et al. [52]	MDA, F2-IsoPs, oxLDL, glutathione
Mason et al. [53]	DCFH, GSSG, GSH, F2-IsoPs, SOD
Wu et al. [54]	F2-IsoPs, SOD, GSH-Px, Hs-CRP, IL-6, TNF-α, MCP-1, MPO, PGE2, LTB4
Oliveira et al. [55]	NADPH oxidase, Zn-SOD, Mn-SOD, IL-6,TNF-α, IL-4, IL-10
Jamilian et al. [56]	TAC, MDA, GSH, hs-CRP, NO

F2-IsoPs, F2-isoprostanes; oxLDL, oxidized LDL-cholesterol; DCFH, 2′,7′-dichlorofluorescin; GSSG, oxidized glutathione; GSH, glutathione; GSH-Px, glutathione peroxidase; TNF-α, tumor necrosis factor α; MCP-1, monocyte chemoattractant protein 1; MPO, myeloperoxidase; PGE2, Prostaglandin E2; LTB4, Leukotriene B4; NADPH, nicotinamide adenine dinucleotide phosphate; IL-4, interleukin 4; IL-10, interleukin 10; TAC, total antioxidant capacity; NO, nitric oxide.

**Table 3:** Effects of Fruit on Inflammation and Oxidative Stress [21,23,57,58,59,60].

Authors	Study Design	All Patients	Type of Fruit	Oxidative Stress and Inflammation Effect
Hedge et al. [21]	Clinical Trial	123	Sweet lime, orange, apple	Reduction in oxidative stress
Hsia et al. [23]	Randomized Controlled Trial	35	Cranberry	Reduction in oxidative stress, no effect on oxidative stress markers
Banini et al. [57]	Randomized Controlled Trial	52	Muscat grape	Reduction in oxidative stress
Hokayem et al. [58]	Randomized Controlled Trial	38	Grape	Reduction in oxidative stress
Sohrab et al. [59]	Randomized Controlled Trial	60	Pomegranate	Reduction in oxidative stress
Nemes-Nagy et al. [60]	Clinical Trial	30	Blueberry	Reduction in oxidative stress

**Table 4:** Summary of the effects of particular hypoglycemic drugs on oxidative stress and inflammation [67,68,70,73,74,75].

Authors	Study Design	All Patients	Drug Class	Effect on Oxidative Stress/Inflammation
Benhamou et al. [67]	Clinical Trial	30	Flexible insulin therapy	No effect on oxidative stress and inflammation
Chakraborty et al. [68]	Randomized Controlled Trial	208	Metformin	Reduction in inflammation and oxidative stress
Singh et al. [70]	Randomized Controlled Trial	60	Metformin and Pioglitazone	Reduction in oxidative stress
Rizzo et al. [73]	Randomized Controlled Trial	90	Sitagliptin, vildagliptin	Reduction in oxidative stress and inflammation
Wu et al. [74]	Randomized Controlled Trial	23	Exenatide	Reduction in oxidative stress and inflammatory markers
Rizzo et al. [75]	Clinical Trial	20	Liraglutide in addition to metformin	Reduction in oxidative stress

## Data Availability

The data used in this article are sourced from materials mentioned in the References section.
